# Learning Probabilistic Features for Robotic Navigation Using Laser Sensors

**DOI:** 10.1371/journal.pone.0112507

**Published:** 2014-11-21

**Authors:** Fidel Aznar, Francisco A. Pujol, Mar Pujol, Ramón Rizo, María-José Pujol

**Affiliations:** 1 Departamento de Ciencia de la Computación e Inteligencia Artificial, Universidad de Alicante, Alicante, Spain; 2 Departamento de Tecnología Informática y Computación, Universidad de Alicante, Alicante, Spain; 3 Departamento de Matemática Aplicada, Universidad de Alicante, Alicante, Spain; Nanjing University of Aeronautic and Astronautics, China

## Abstract

SLAM is a popular task used by robots and autonomous vehicles to build a map of an unknown environment and, at the same time, to determine their location within the map. This paper describes a SLAM-based, probabilistic robotic system able to learn the essential features of different parts of its environment. Some previous SLAM implementations had computational complexities ranging from *O*(*N*log(*N*)) to *O*(*N* ^2^), where *N* is the number of map features. Unlike these methods, our approach reduces the computational complexity to *O*(*N*) by using a model to fuse the information from the sensors after applying the Bayesian paradigm. Once the training process is completed, the robot identifies and locates those areas that potentially match the sections that have been previously learned. After the training, the robot navigates and extracts a three-dimensional map of the environment using a single laser sensor. Thus, it perceives different sections of its world. In addition, in order to make our system able to be used in a low-cost robot, low-complexity algorithms that can be easily implemented on embedded processors or microcontrollers are used.

## Introduction

In recent years, there has been an increasing demand for autonomous systems able to characterize, quantify and cope with complex environments. The need arises from several key areas such as industrial automation, architecture, agriculture or construction, among others [1]. One common feature for all these applications is that they require three-dimensional data to model the environment. The modelling process can be completed by mobile robots with laser sensors for generating three-dimensional data from the environment when performing different tasks. Even in areas such as mining and searching for survivors when a natural disaster occurs, it could be convenient that small robots work with this technology. However, it is a complex problem that cannot often be developed in real time.

A robot needs to know where it is located within its environment in order to be able to move autonomously. Self-localization requires the existence of a map and it has led to the development of algorithms for Simultaneous Localization and Mapping (SLAM) over the past 20 years, where the robot builds parts of a map as it explores the environment [2]–[6].

The most common algorithm for SLAM is the stochastic SLAM [7], [8]. This method explicitly takes into account the errors made in the measures. These errors generate uncertainty in the estimation of the features of the environment and, consequently, they result in uncertainties in the position of the robot. Therefore, the map of the environment and the robot's position are directly related. Stochastic SLAM implementations usually represent the uncertainties and correlations using Gaussian distributions (or probability density functions, PDF) and propagate the uncertainties using the Extended Kalman Filter (EKF). These implementations are generally known as EKF-SLAM [9]–[11]. However, one of the main problems with EKF-SLAM is that it requires having geometric models of the environment, which limits its use to environments where such models are available.

An alternative to these models are the so-called scan-correlation procedures [12], [13], where the maximum alignment between two sets of data is estimated. Then, given a series of observations and a reference map constructed also from raw data, the robot self-locates without a prior conversion of the data. Consequently, the observations are simply aligned with the data in the map to maximize the correlation of the measures. Scan-correlation has been used as a location algorithm for a priori maps and the most commonly used methods are the Iterative Closest Point (ICP) algorithm [14]–[16] and the correlation matrix of occupancy grids [12], [13], [17]–[19].

In recent years, the interest of researchers has focused on reducing the computational cost of performing SLAM in large environments [20]. Some processes associated to the move-sense-update cycle of EKF-SLAM are linear in the number of map features *N*: vehicle prediction and inclusion of new features [21], [22]. Because of the correlations between all pair of map features represented by the covariance matrix, the size of its entries grows as *O*(*N* ^2^). Consequently, EKF-SLAM techniques have an important limitation: the computational cost of updating the map at each step is of order *O*(*N* ^2^).

In addition, the generic implementation of ICP algorithm has also a complexity *O*(*N* ^2^). There are some possible modifications to achieve high-speed ICP algorithms, such as using closest-point matching and a point-to-point error metric [23]. Although ICP and its extensions produce good results, they are only guaranteed to converge towards a local minimum and may not always find the correct transformation [24]. Furthermore, these algorithms suffer from computational complexity problems when dealing with large scale environments because the point to point association rules they use result in a *O*(*N*log(*N*)) complexity in the best case. Moreover, the optimizations generally require a high memory cost and, therefore, they are not appropriate for low memory devices such as an embedded robotic system.

Consequently, this work aims at developing a robot system able to learn a set of features from a complex environment for autonomous navigation. Afterwards, the robot identifies those areas similar to the previously learned features as it moves through the environment. At the same time, it generates a three-dimensional map of the environment.

The algorithm is designed to have a reduced computational complexity in order to be applied to low performance embedded systems, minimizing, as a result, both cost and power consumption. Thus, we propose to use only a laser sensor to minimize both the cost and the size of the robot. Since SLAM implementations are usually computationally expensive, an iterative reconstruction algorithm for the map is considered. As Thrun said [25], these methods are fast and can be applied successfully to real-time applications provided that it is not necessary to get a perfect global map in cyclic environments. The results of the experiments using our model show that the computational complexity of our approach is reduced to *O*(*N*).

A revision of recent approaches and a new proposal for iterative reconstruction methods will be discussed next.

## Methods

### Iterative reconstruction of maps by SLAM

In the literature, a map *m* is defined as a set of readings and their positions [26], [27]. This way, a map *m* is established as *m_t_* = {*s_t_*, *l_t_*}, where *s_t_* = (*x*, *y*, *θ*)*_t_* is the position of the robot at time *t* and *l_t_* =  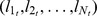 represents the vector of *N* sensor readings at the same moment. Let *L* be the set of all the possible readings, such that 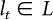.

The purpose of the mapping process is to obtain the map that optimizes the representation of a dataset [28], [29]. The traditional model of incremental mapping makes use of the previously calculated odometry and the laser readings to obtain the most likely robot's current position. With this prediction, the current position is recalculated by repeating the process until the construction of the map is finished. Therefore, the most likely position 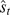 at time *t* is obtained by using a conditional probability as follows: 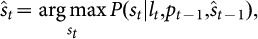
(1)where *s_t_* is the position, *l_t_* is the vector of sensor readings, *p_t−_*_1_ is the calculated odometry and 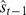 is the estimated position at the previous timestep, as defined in the paragraphs above. Once the position is obtained, the map *m_t_*_+1_ is updated accordingly:
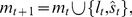
(2)where *m_t_* is the map calculated at time *t*.

There are several approaches to solve Eq. (1). The most usual solution is to apply the Iterative Closest Point (ICP) algorithm or some of its variants [14] to estimate the correspondence between the positions of the robot at different times. This method matches points of both scans by finding for each point of the first scan the nearest point in the other one. Afterwards, a motion vector is estimated to cope for their misalignment. However, the algorithm has a complexity *O*(*N^2^*) and there are cases in which a sudden change in the environment results in an incorrect reading. Consequently, the error will be spread over the map.

There is another algorithm, called Random Sample Consensus (RANSAC), widely used in the area of artificial vision, which makes a robust adjustment of a model to a given dataset. However, its main drawback comes from the fact that it does not take into account that the points obtained from a reading have a certain order, so it does not achieve accurate results for this kind of applications [30]. There have been a number of recent efforts aimed at increasing the efficiency of the basic RANSAC algorithm [31]. Some of the strategies [32] aim to optimize the process of model verification, while others [33] modify the sampling process in order to preferentially generate more useful hypotheses. While these efforts are promising, there are still problems to implement this technique when either low performance or low memory resources are required.

In our particular case, several assumptions can be made. First of all, a scenario where a robot moves through a corridor in an office environment is considered. Then, it is assumed that the robot moves parallel to the walls in the corridor, thus simplifying the correspondence between the current and the previous readings. The assumption is relatively easy to accomplish by fusing the readings obtained from both the laser and the ultrasonic sensors with the odometry of the robot. A model to fuse any kind of information using the Bayesian paradigm was proposed by Aznar et al. [34]. Using this model, a physical agent develops a generic task working under uncertainty. Moreover, the system allows the detection of any failures in the sensorial system, thereby detecting if any sensor is returning erroneous readings.

Furthermore, suppose that the distance between a position and the next reading is small enough to consider that the distribution *P*(*s_t_*|*l_t_*, *p_t_*_−1_,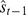) = *G*(*μ*, *σ*) has a Gaussian shape. In addition, assume that given the current readings and the previous position estimation, the system selects (or *prefers*) a certain location over the others (although there are also some other likely places around this location). As a result, in order to compute the Gaussian distribution two parameters are needed: its mean and its variance. Consequently, for all the readings the average movement of the robot can be calculated as a weighted mean of the movement estimated from the current and the previous readings. Thus, the mean of this distribution μ is defined as:
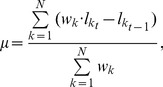
(3)where *w* = (*w*_1_, *w*_2_, …, *w_N_*) is a function of the variance of each point in the current reading. Using Eq. (3) the variance σ^2^ of the distribution is obtained as:
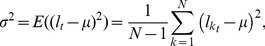
(4)where E(*X*) is the expected value of *X*.

Assuming that the movement of the robot remains constant throughout the area where it is located, then the correspondence between the current reading and the previous map of the world can be obtained. However, this assumption is not always true, since the continuity cannot be ensured when moving through different sections within the environment. Nevertheless, it can be still assumed that there is a set of common features that remain unaltered between a measure and the following one. For instance, although unexpected elements could appear on the right side of a corridor between a measure and the following one, the left wall may not have changed, making it a common area for both readings. Consequently, Eq. (3) can be used as a preliminary estimation for the system.

As a result, this estimation is calculated first and, afterwards, the system obtains the elements that have changed between the two measures. Once these points are known, their weight (i.e., their variance) is updated. Therefore, the elements with the highest probability of having changed are assigned a lower weight when making the correspondence. In addition, as a model of the movement of the robot throughout the environment is obtained, the maximum displacement between two measures is limited. Finally, for all the points, the new weight and the initial one are combined.

Our proposed algorithm is described next:

- The inputs to the algorithm are:
  - the weight of the current reading elements, 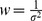,
  - the points in reading *l_t_*, and
  - the points to be adjusted in the previous reading, *l_t−_*_1_.
- Calculate the estimated distribution parameters, that is, the estimated mean 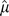 and the estimated variance 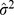, as follows:
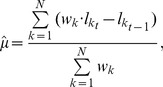


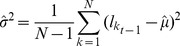
(5)
- Recalculate the reading position using 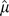, and recalculate the weight for each of the points, 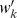, using the following expression:
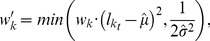
(6)where 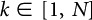.
- Calculate the final parameters of the distribution using Step (ii) with the new weights 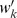.

Consequently, after applying the algorithm the distribution *P*(*s_t_*|*l_t_*, *p_t_*_−1_, 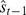) can be approximated and, as a result, the most likely location of the robot at time *t* is obtained. This approach has several advantages over previous works: first, for any reading it allows to set the weight of any point. Thus, the positions with incorrect matches can be removed, making the system robust against interferences. It also reduces the complexity to *O*(*N*), where *N* is the number of points obtained by each reading of the laser. Finally, the maximum range of the robots displacement in a period of time can be introduced and taken into account for the matching process.

### A Proposal for Learning Features

Once our algorithm has been defined, the design of an application that can extract distinctive features of the environment, such as a door, a window or free space, will be discussed next.

Although the localization of automatic landmarks for robot navigation is a well-known (yet difficult) task, in this work it is supposed that the user teaches the robot which features are useful in a particular environment. The assumption has several advantages: firstly, it allows different tasks to be executed using only one system, without having to reprogram the robot. Thus, the same application can be used for different environments. Moreover, this teaching process minimizes errors, while maximizes the robots adaptation to environments with uncertainty.

Therefore, the user first teaches the robot a set of readings *C^ϕ^* ⊂ *L* that represent a certain feature *ϕ*. Given a new map *m_t_* obtained from a new reading from the laser sensors of the robot, the posterior probability of the set of readings *C^ϕ^* given *m_t_*, *P*(*C^ϕ^*|*m_t_*), is the probability that the map *m_t_* belongs to the set of readings *C^ϕ^*:
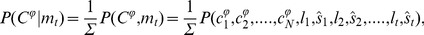
(7)where 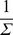 is a normalization constant and *N* is the number of readings that define feature *ϕ*. This distribution can be easily calculated by obtaining the probability of each of the readings of feature *ϕ*, 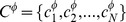, given a particular reading *l_k_* (which is also related to position *s_k_*).

The probability of making an error when classifying the current readings *l_t_* of map *m_t_* into the learned readings *C^ϕ^* can be computed as [35]:
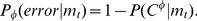
(8)


Consequently, the minimum error probability for a given map *m_t_* is:
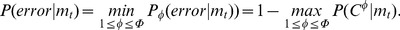
(9)where ϕ is the number of classes in the learned features. From this expression, the error probability is minimized if we classify the current readings *l_t_* of map *m_t_* in the class with the highest posterior probability.

Thus, the process followed to learn a particular feature is the following one:

- The set of *N* readings *C^ϕ^* determining a feature is obtained.
- Each reading consists of a series of points which compute, for any given point, the distance from the center of the robot to an obstacle. It is assumed that the reading points have a Gaussian distribution. Recall that when using our iterative adjustment algorithm, the variance of each of the reading points is known.
- For each of the angles in all the features, a Gaussian distribution is obtained. That is, a set of 180 Gaussian distributions (a Gaussian per angle) are obtained, which will define feature *ϕ*.
- Using Eq. (7), the probability of a given reading *l_t_* to have been generated by feature *ϕ* is calculated.

### Test environment design

The experimental process carried out in order to test our algorithm and the datasets used to complete the experiments is described now.

**Figure 1:**
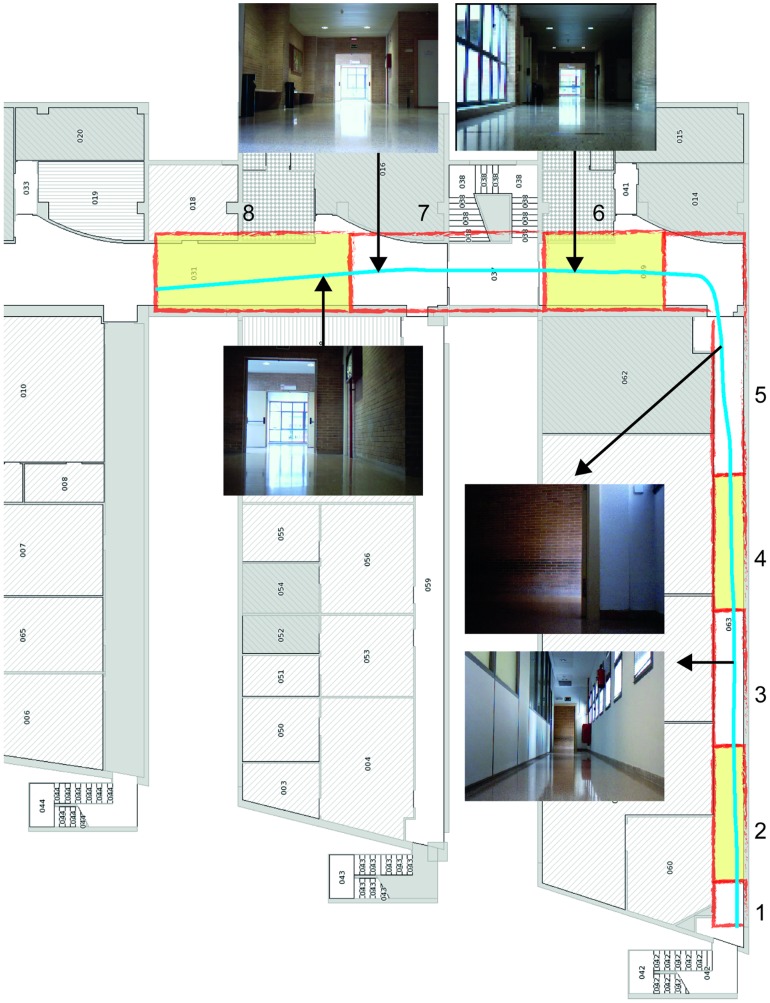
Map of the corridor where the tests have been carried out. Some pictures of selected areas in the corridor are shown, as well as an example of the path followed by the robot to acquire the laser readings.

First, to test the performance of our alignment algorithm, real laser readings obtained from one of the corridors in our research laboratory were used. The data are publicly available at http://www.i3a.ua.es/public/PolitecnicaII_LaserData052013. The corridor has some curved walls and a great amount of windows and columns that make it difficult to obtain readings using active sensors, such as the laser sensor used to collect data. Therefore, it is an appropriate real environment to test our system, since two consecutive readings, although sharing some common areas, may have substantial differences. A map and photographs of the test area are shown in Fig. 1.

Furthermore, to estimate the complexity of our reading alignment algorithm, a set of random point clouds -ranging from 100 to 10,000 points- has been used. Afterwards, a Gaussian noise was added to the point clouds. The point clouds were employed to test both our algorithm and an optimized implementation of ICP, which used a buffer of kD trees to improve the speed of the algorithm (see [36] for details). Using these data, the alignment algorithm was tested and the 3D environment in which a robot navigates was reconstructed.

In addition, the data readings were acquired by a Pioneer DX robot. The robot used only a SICK LMS-200 laser sensor [37]. This sensor swept a laser ray in a horizontal plane. It gave the distance to each obstacle in its range of view, giving distance measurements over a 180° area, up to 80 meters away. Afterwards, the observed 3D data were matched to the previously learned model of the environment.

**Figure 2:**
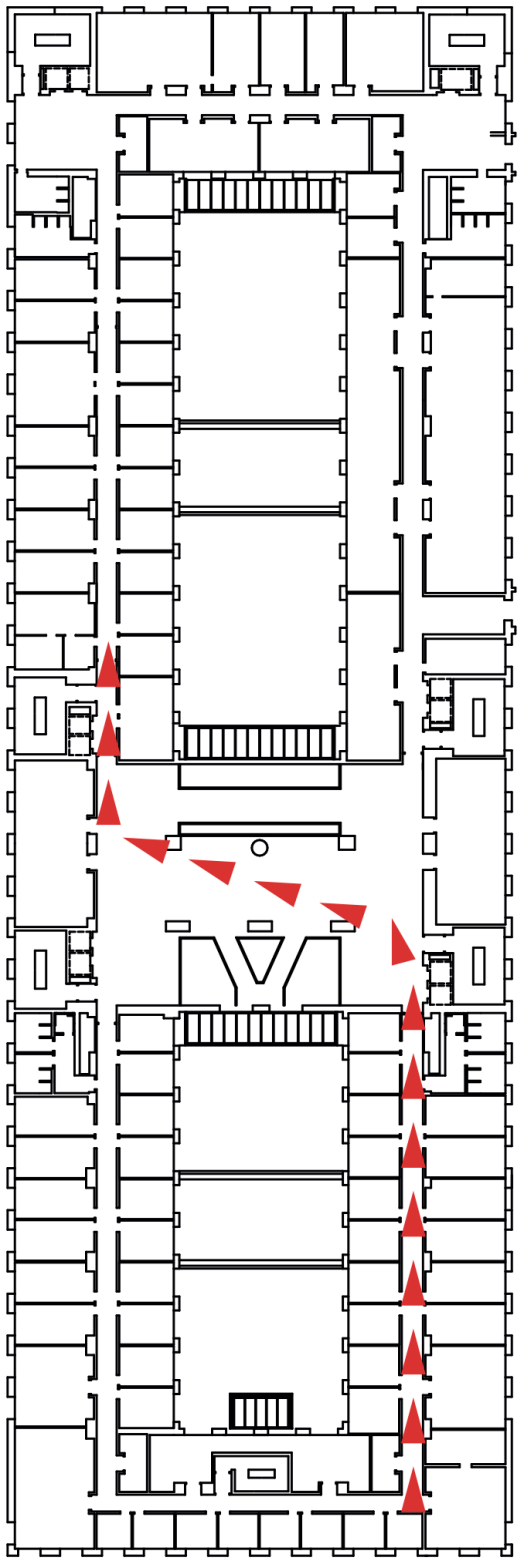
Map of a corridor from the Rawseeds project. It belongs to building A from the Università di Milano-Bicocca (Italy). This environment includes narrow corridors, doors or staircases. The path followed by the robot is marked in red.

Finally, our algorithm was evaluated on the real data given by an indoor dataset taken from the Rawseeds project [38]. In particular, the subsequence of the Bicocca 2009-02-27a dataset from the Rawseeds dataset was used. The dataset was static (i.e., did not include moving objects such as people) and had natural lighting; the data from building A, belonging to the Università di Milano-Bicocca, in Milan (Italy), were selected. In this building one can find a wide range of different of architectural features, such as narrow corridors, columns, staircases and elevators. In Fig. 2 the map of the building and the robot path for the dataset are shown.

After defining the methods, the results of our experiments will be shown next.

## Results and Discussion

In this section, the main results in terms of feature learning and map reconstruction using a laser sensor are reported. To begin with, our method was experimentally verified and compared with a state-of-the-art version of the ICP algorithm. Then, different tests on map reconstruction and feature learning are shown in order to verify the suitability of our approach. After the experiments, the speed of our algorithm with respect to the ICP approach was obtained. Finally, a practical validation of the assumptions we made to develop both the alignment method and the feature learning process was completed.

### Comparison between methods

In this section a comparison between our proposed method for the iterative reconstruction of maps and the optimized ICP algorithm described in [36] is made. As mentioned before, one of the main drawbacks of ICP comes from unexpected changes in the environment that can lead to wrong sensor readings.

**Figure 3:**
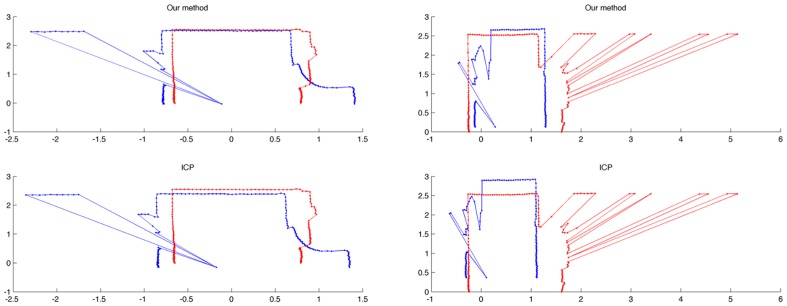
Two correspondences between readings from different sections using our method and the ICP algorithm. On the left, the alignment of two close areas for both our method and the ICP is shown. On the right, the alignment of two distant areas is considered. The blue lines represent the readings from the original section, whereas the red lines represent the readings from the next section to be aligned.

In our particular case, this fact causes that some sections in the environment do not have continuity between them. As a result, some experiments were performed to compare the correspondences made by our approach and the ICP algorithm for some sections of the corridor in Fig. 1. The sections were selected so that they are not continuous; thus, there are some elements, such as windows and doors, which make it difficult to align the sections correctly. From the results of the experiments (see Fig. 3) it can be seen how our approach works in a proper way by providing a consistent alignment, which is similar to the alignment given by the ICP algorithm. However, as shown later, the execution time for our method is substantially lower. The alignment of two close areas for both our method and the ICP is shown in Fig. 3(a), whereas the alignment of two distant areas can be seen in Fig. 3(a). In both cases, the blue lines represent the readings from the original section and the red lines represent the readings from the next section to be aligned.

Consider now that the corridor in Fig. 1 is divided into the 8 sections. For each section, 250 readings from the laser sensor have been obtained. As mentioned before, the data can be found at http://www.i3a.ua.es/public/PolitecnicaII_LaserData052013. The alignment of the optimized ICP algorithm against our approach was compared by using data collected from 5 different trials for each section. Table 1 shows both the mean alignment error and the standard deviation for each area. The computing times to process all the readings for each section of the corridor are also shown for both algorithms. The tests have been performed using Matlab with a Mac Book Air (1.8 GHz Intel Core i5 and 8 GB memory).

**Table 1:** Errors and computing time analysis for our method and the ICP algorithm.

	Our method			Optimized ICP		
Section	Mean error (m)	Standard deviation (m)	Computing time (s)	Mean error (m)	Standard deviation (m)	Computing time (s)
1	0.43	0.83	0.42	0.43	0.83	3.82
2	0.41	1.00	0.42	0.41	1.00	3.82
3	1.33	2.93	0.42	1.33	2.92	3.84
4	0.27	0.43	0.42	0.28	0.44	3.84
5	1.41	2.28	0.42	1.46	2.44	3.88
6	0.28	0.63	0.41	0.29	0.66	3.87
7	2.83	3.18	0.42	168.37	197.48	3.86
8	0.35	0.77	0.42	22.45	101.78	3.86

From Table 1, it can be noticed that in most cases the error made by our algorithm is almost the same as the one in ICP, except for some areas (such as sections 7 and 8) where the alignment of ICP was wrong and led to a higher alignment error in that area. Thus, it is shown that our initial assumptions about using Gaussian distributions to model the probability between the distance about the current position and the next sensor reading was correct. As it was expected, they gave similar results to the optimized ICP approach.

Regarding the computing times, our algorithm proved to be much faster than the ICP method, even when the optimized version with kD trees was used (about 10 times faster for the overall process of mapping the corridor). Moreover, the execution did not involve storing any complex data structure with special memory requirements. However, the ICP version with kD Trees, although it was faster than the original ICP, required the use of a tree, which is costly in terms of memory. Additionally, in real applications of robot systems, satisfying the requirement of computing time limitation is even more important than reducing the algorithm complexity. This fact could be especially helpful when designing a low-cost robot with low memory resources, such as an embedded processing unit, where the constraints of a low-cost consumer product pose a major challenge for developing reliable localization systems.

**Figure 4:**
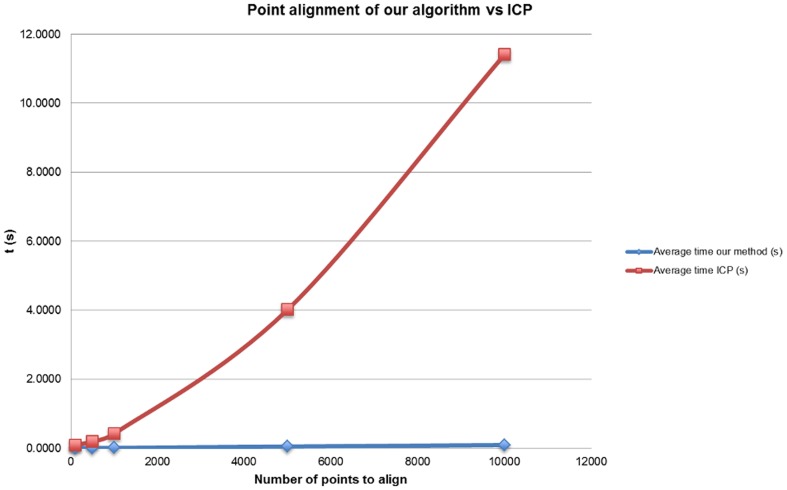
Average run time for our approach and the ICP optimized algorithm. The point clouds are random and range from 100 to 10,000 points.

**Figure 5:**
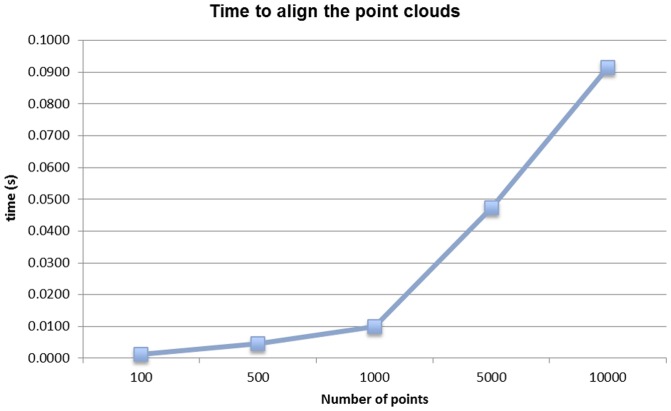
Average run time for our approach.

In order to verify the computational complexity of our algorithm, an alignment test using 3D random points was performed. Thus, several point clouds were generated, using 100, 500, 1,000, 5,000 and 10,000 points, where each coordinate point was between 0 and 100 and a Gaussian noise was added in order not to achieve exact alignments. The execution time for each of the algorithms was calculated, for 10 different trials. The results are shown in Table 2 and, graphically, in Fig. 4. An enlarged view of the time results for our approach is shown in Fig. 5.

**Table 2:** Alignment using 3D random point clouds: errors and computing time analysis.

	Our method			Optimized ICP		
No. of points	Mean error (m)	Standard deviation (m)	Computing time (s)	Mean error (m)	Standard deviation (m)	Computing time (s)
100	17.97	1.66	0.00	17.08	2.03	0.09
500	39.94	2.22	0.00	42.05	2.37	0.20
1,000	55.38	3.73	0.01	58.90	4.33	0.42
5,000	128.21	2.30	0.05	133.65	3.01	4.02
10,000	181.58	3.00	0.09	189.64	4.46	11.41

As it can be noticed when comparing the execution of both algorithms, the running time of our algorithm manifested a linear trend with a complexity of *O*(*N*), which was an order of magnitude faster than the implementation of ICP using kD trees (with complexity *O*(*N* ^2^)). Moreover, our approach required low memory usage as no additional data structures were needed. Additionally, the alignment of the data had approximately the same error in both algorithms. Thus, although the ICP algorithm achieved the global optimum after running a sufficient number of iterations, our method obtained similar results when using both real data from the laser readings and several random point clouds.

### Tests on Reconstruction

**Figure 6:**
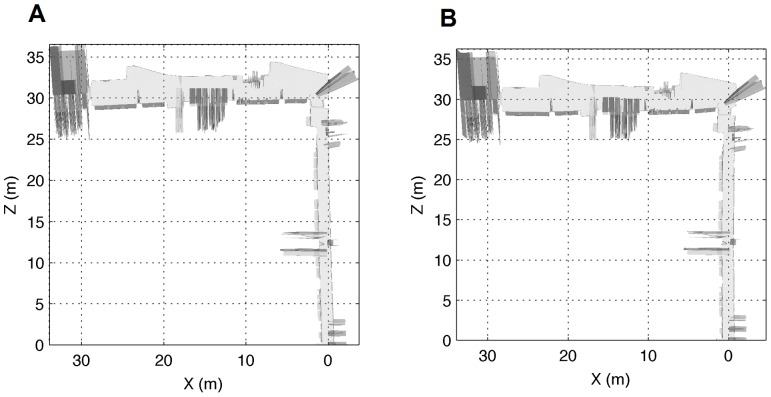
Different approaches for reconstructing a map of the environment. (a) Map of the corridor from our department (see Fig. 1) generated only with odometric information. (b) Map generated using the SLAM method proposed in this paper.

Some tests on map reconstruction for different environments are considered next. The map of our corridor reconstructed only using the odometry of the robot and the same map reconstructed using the SLAM algorithm based on our incremental adjustment are shown in Fig. 6. This test area has been specially selected because it contained a standard corridor section (where the two walls were parallel) and another section where the right wall was progressively curved. Thus, the map of the corridor in Fig. 1, when generated only with odometric information, unboundedly accumulated errors due to different wheel diameters, wheel-slippage, wheel misalignment, and finite encoder resolution [39] (see Fig. 6(a)).

When the same map was generated using our SLAM approach, the algorithm created a straight corridor (see Fig. 6(b)), instead of a slightly curved corridor as in Fig. 6(a). Specifically, in Fig. 6(a), the reconstruction of the corridor shows a progressive rotation from position (0,0) to position (0,30), due to the errors accumulated by the odometry of the robot. Our algorithm corrected this rotation in the reconstruction and removed it, as shown in Fig. 6(b). In addition, notice that the upper section did not contain any parallel walls, which could cause some wrong local correspondences that must be corrected to generate the global map. Nevertheless, the map obtained was representative enough for localization tasks, even if the robot was moving through the upper corridor.

**Figure 7:**
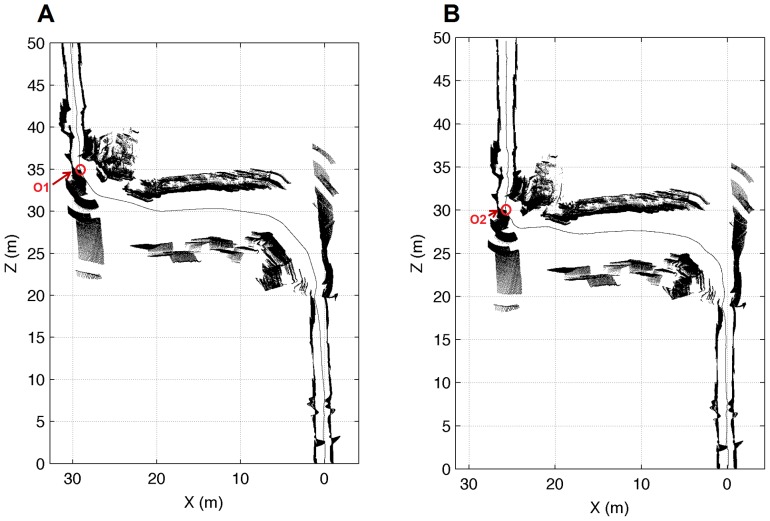
Reconstruction of a map from the Rawseeds project. (a) Reconstruction generated only with odometric information. (b) Map reconstructed using our approach.

Some reconstruction tests have been also performed using the public data from the Rawseeds project and taking into account the environment shown in Fig. 2. In this case, some challenging elements, such as narrow corridors, windows and staircases, can be found. Thus, the reconstruction using only the odometric information and the representation of the results after applying our method are graphically described in Fig. 7(a) and 7(b), respectively. As in Fig. 6, the reconstruction of the upper corridor had a progressive rotation when the odometry of the robot was only used (see Fig. 7 (a)). On the contrary, Fig. 7(b) shows how our algorithm corrected the odometric errors and achieved a much better reconstruction of a straight corridor. Moreover, the origin of the upper corridor differs greatly depending on which approach was considered. Thus, if the odometry was used to reconstruct the map, the origin of the upper corridor was located at position O1, as shown in Fig. 7(a), which approximately corresponded to position (29, 35). When using our method, this origin was located at position O2, corresponding to position (26, 30) in Fig. 7(b), which resulted in a much more accurate reconstruction of the original corridor. Consequently, our approach was able to improve the reconstruction of the environment again when compared to using only the odometry.

From the results, notice that our approach created consistent local maps. The consistency could not be guaranteed for global maps, though, because our algorithm did not perform correlations between readings. However, for the initial phase of the learning task it was not required to have a global map, since it could be obtained by relocating the features to be learned as the map was being built. As a result, the computational requirements of the algorithm were initially reduced while providing, at the same time, reliable maps for our learning task.

### Tests on Feature learning

This section describes how the feature learning process for our robot system operates. There are multiple applications of this process. Thus, it can be defined as a practical task for the robot to navigate as quickly as possible into a goal area. The area is not marked to other parts of the environment. Therefore, a feature learning stage is required to get to the desired area. Different behaviors related to these features can be also defined to enable the robots to navigate through an environment, such as “going along a corridor”, “going around a corner”, “going towards a landmark” or “stopping”.

**Figure 8:**
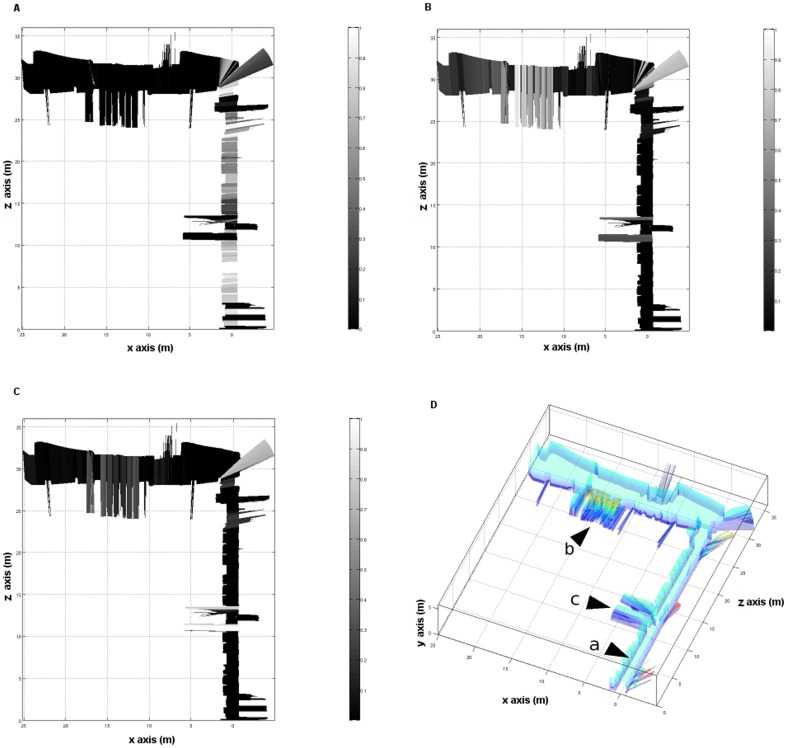
Example of localization using three different features. The membership of the trained feature for all the positions in the map is shown, where black colors indicate that the property does not belong to the trained feature. (a) Training using a corridor area with small windows (windows type A). (b) Training in an area with big windows of the upper corridor (windows type B). (c) Training with an ‘open door’ feature. (d) 3D generated map, where the training positions are marked.

Firstly, the environment in Fig. 1 was considered, where two sections of a corridor were found, with different elements (or *features*), such as windows, doors and columns, located throughout the environment. The robot was assumed to have learned three different features: a window type A, which is a small window at the top of a corridor (as shown in section 3 of Fig. 1), a window type B, which is a big window in the middle of a corridor (as shown in section 6 of Fig. 1), and an open door. To complete this process, a set of 10 laser readings for each feature were used. Then, the robot tried to identify the learned features as it navigated throughout the environment. Fig. 8 shows the results on the location of the three features on the map, using the algorithm previously described. Thus, a windows type A is trained (see Fig. 8(a)). Then, the robot was taught the feature ‘windows type B’ (see Fig. 8(b)). Finally, the robot learned the characteristics belonging to an open door (see Fig. 8(c)).

As it can be seen, once the system was taught, it was possible to know the probability that a given area belonged to the trained feature. For instance, the robot had been trained to locate a window type B, by taking 10 readings that were close to the position ‘b’ in Fig. 8(d). Therefore, the probability that a particular area in the map would be a window type B is shown in Fig. 8(b). It can be seen how the window area in the upper corridor had a high probability (over 0.7), as well as the areas where the readings didnt find any obstacles for over 6 meters, such as the corner between both corridors. Similarly, the open door in training position ‘c’ had a probability of 0.35 of being a window, due to the similarity between the measures. For all the other areas, the probability was close to 0. This fact can be extended to any other feature needed to be learned.

**Figure 9:**
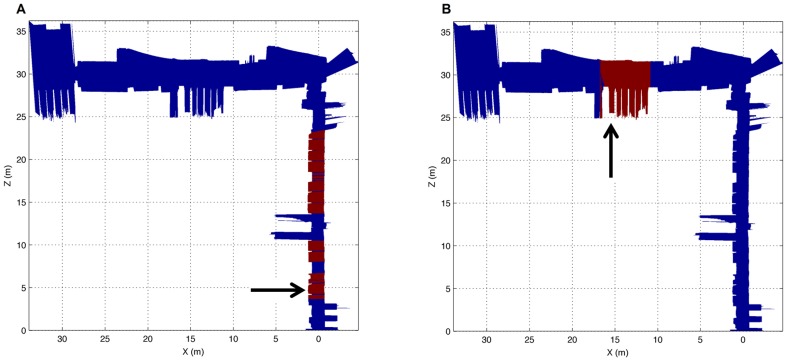
Feature learning for the corridor in **Fig. 1**. (a) Results after the system has been trained to learn a ‘window type A’ feature. (b) Results after the system has been trained to learn a ‘window type B’ feature. Sections in red indicate the trained feature has been found and sections in blue that this feature has not been detected. The arrows point to the position from where the 10 laser readings were acquired and the rest of the map has been used as the test set.

To complete our tests, several experiments have been carried out by using a reduced training set with only 10 readings for each feature. The feature learning process has been tested for both our real environment and for the map from the Rawseeds project in Fig. 2. On the one hand, the system was trained in order to locate the windows type A and B from our corridor. Both types of windows were accurately located, despite having only used a training set of 10 readings for each feature, as shown in Fig. 9. In both cases, sections in red indicate that the trained feature has been found and sections in blue show that this feature has not been detected. The arrows point to the positions from where the 10 laser readings were acquired and the rest of the map has been used as the test set. Taking into account the obtained results, the localization of both features was correct.

**Figure 10:**
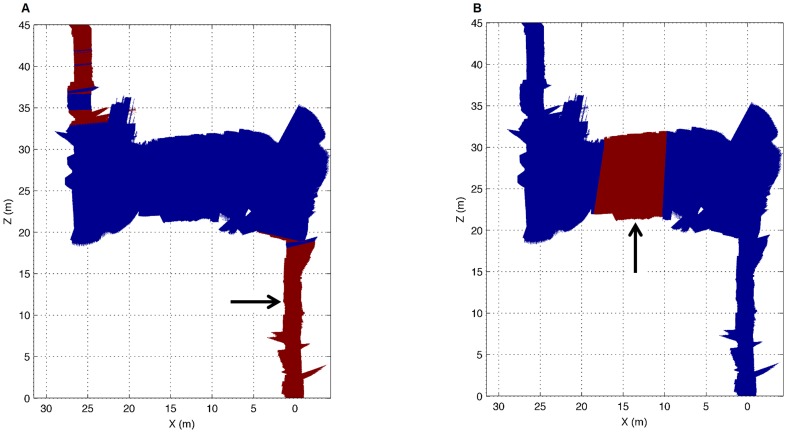
Feature learning for the corridor from the Rawseeds project. (a) Results after the system has been trained to learn a ‘corridor’ feature. (b) Results after the system has been trained to learn a ‘hall’ feature. Only 10 laser readings were acquired from the positions indicated by the arrows. The rest of the map has been used as the test set.

Finally, several tests to locate some features, such as a corridor area and a hall area, using the reconstructed map from the Bicocca dataset were also performed. Only 10 laser readings were used for the training process of each feature. As in the previous example, the results of the teaching process were adequate and verified that our approach achieved satisfactory classification accuracy, especially for the corridor area, as shown in Fig. 10(a). The hall area was an area with many obstacles, columns, tables and staircases. That is the reason why only a portion of the hall was identified as the trained feature (see Fig. 10(b)). Consequently, the classification process not only worked correctly, but it also provided information about each of the trained features, and they can even be compared. Thus, since our approach works with a probability distribution, the likelihood of classifying an area as a certain feature can be easily calculated.

After completing all the experiments, it became clear that our method achieved similar accuracy than related previous methods, while requiring lower computational processing. As a consequence, it is found that our assumptions were correct and that our approach could be used as an alternative to traditional SLAM-based algorithms for low memory robotic platforms.

## Conclusions

This paper has presented a system capable of locating features by making a previous training in a three-dimensional map generated by SLAM. The robustness of the algorithm for non-cyclic mapping environments has been shown. After introducing the proposed system, a set of experiments for locating features have been run. The information from only one laser sensor has been utilized and the correct location of several landmarks, such as open doors and windows, has been shown. Obviously these initial tasks can be used as a basis for conducting more complex services, such as detecting defects in construction or monitoring industrial systems.

As a future work, the proposed method can be completed by optimizing the global map used for the localization of features, in order to use it in cyclic environments. Moreover, it would be convenient to add an omnivision camera to fuse the three-dimensional map information with the obtained images from the camera. Thus, by segmenting the images and fusing them with the segmentation information obtained from the laser, the location of specific features will be improved.
