# Correction: Learning Probabilistic Features for Robotic Navigation Using Laser Sensors

**DOI:** 10.1371/journal.pone.0119342

**Published:** 2015-03-23

**Authors:** The PLOS ONE Staff

The following information is missing from the Funding section: This work was also supported by the Spanish Ministerio de Economía y Competitividad, project TIN2013-40982-R.
